# Cerebello-cerebral connectivity in the developing brain

**DOI:** 10.1007/s00429-016-1296-8

**Published:** 2016-08-29

**Authors:** Kay Pieterman, Dafnis Batalle, Jeroen Dudink, J-Donald Tournier, Emer J. Hughes, Madeleine Barnett, Manon J. Benders, A. David Edwards, Freek E. Hoebeek, Serena J. Counsell

**Affiliations:** 1 Division of Imaging Sciences and Biomedical Engineering, Centre for the Developing Brain, King’s College London, London, SE1 7EH UK; 2 Department of Neonatology, Erasmus Medical Centre, Sophia Children’s Hospital, Rotterdam, The Netherlands; 3 Department of Radiology, Erasmus Medical Centre, Rotterdam, The Netherlands; 4 Department of Neuroscience, Erasmus Medical Centre, Rotterdam, The Netherlands; 5 Division of Imaging Sciences and Biomedical Engineering, Department of Biomedical Engineering, King’s College London, London, SE1 7EH UK; 6 Department of Perinatology, Wilhelmina Children’s Hospital and Brain Center Rudolf Magnus, University Medical Center Utrecht, Utrecht, The Netherlands

**Keywords:** Infant, Brain, Cerebellum, Diffusion MRI, Tractography

## Abstract

Disrupted cerebellar development and injury is associated with impairments in both motor and non-motor domains. Methods to non-invasively characterize cerebellar afferent and efferent connections during early development are lacking. The aim of this study was to assess the feasibility of delineating cortico-ponto-cerebellar (CPC) and cerebello-thalamo-cortical (CTC) white matter tracts during brain development using high angular resolution diffusion imaging (HARDI). HARDI data were obtained in 24 infants born between 24^+6^ and 39 weeks gestational age (median 33^+4^ weeks) and scanned between 29^+1^ and 44 weeks postmenstrual age (PMA) (median 37^+1^ weeks). Probabilistic tractography of CPC and CTC fibers was performed using constrained spherical deconvolution. Connections between cerebellum and contralateral cerebral hemisphere were identified in all infants studied. Fractional anisotropy (FA) values of CTC and CPC pathways increased with increasing PMA at scan (*p* < 0.001). The supratentorial regions connecting to contralateral cerebellum in most subjects, irrespective of PMA at scan, included the precentral cortex, superior frontal cortex, supplementary motor area, insula, postcentral cortex, precuneus, and paracentral lobule. This study demonstrates the feasibility of assessing CTC and CPC white matter connectivity in vivo during the early stages of development. The ability to assess cerebellar connectivity during this critical developmental period may help improve our understanding of the role of the cerebellum in a wide range of neuromotor and neurocognitive disorders.

**Electronic supplementary material:**

The online version of this article (doi:10.1007/s00429-016-1296-8) contains supplementary material, which is available to authorized users.

## Introduction

The cerebellum has traditionally been considered to be involved in motor coordination (Evarts and Thach 1969). However, the role of this structure in non-motor functions such as cognition, emotion and behavior has recently become evident (Buckner, 2013; Schmahmann and Caplan 2006; Schmahmann and Pandya 2008; Tavano et al. 2007; Timmann et al. 2010). In infants, cerebellar lesions may lead to impairments in non-motor as well as motor domains (Law et al. 2011; Limperopoulos et al. 2007, 2009; Tavano et al. 2007; Volpe 2009). The precise pathophysiological mechanisms underlying these deficits are unknown and it remains unclear whether functional impairments in non-motor domains are a direct consequence of impaired cerebellar development and function, or emerge from disrupted cerebello-cerebral connectivity resulting in impaired cortical development.

Volumetric and epidemiological studies have provided evidence for a potential role of the latter mechanisms during early life (Limperopoulos et al. 2005c; Srinivasan et al. 2006), but a detailed characterization of white matter connectivity between cerebellum and cerebrum during the early stages of development is lacking. Using ex vivo high angular resolution diffusion imaging (HARDI), Takahashi and colleagues were recently able to identify coherent white matter pathways forming the inferior, middle and superior cerebellar peduncles in postmortem human cerebellar specimens ranging from 17 to 38 weeks gestational age (Takahashi et al. 2014). These findings indicate that, during the third trimester of pregnancy, the growth rate of the cerebellum is high (Chang et al. 2000; Volpe 2009), and of particular relevance here, that the cerebello-thalamo-cortical (CTC) and cerebro-ponto-cerebellar (CPC) tracts are maturing. These findings may be particularly important for infants born preterm, before 37 weeks of gestation, as lesions affecting the cerebellum are commonly observed during this critical period (Limperopoulos et al. 2005a; Steggerda et al. 2013; Zayek et al. 2012). In addition, abnormalities in the development of cerebro-cerebellar connections are implicated in autism spectrum disorders (ASD) (Marko et al. 2015; Wegiel et al. 2014) and attention deficit hyperactivity disorder (ADHD) (Tomasi and Volkow 2012; Wang et al. 2013). As such, there is a clear need for a tool that allows non-invasive assessment of cerebellar afferent and efferent maturation following preterm birth and in infants considered high-risk for neurocognitive and behavioral disorders.

Diffusion MRI (dMRI) allows white matter structure to be characterised by mapping water molecular motion in tissue. dMRI tractography delineates the trajectories of white matter fibres and enables tract-specific measures to be obtained, allowing comparison of corresponding fasciculi between individuals. The most commonly used approach to analyse dMRI data is the diffusion tensor (DT) model. However, DT approaches to study white matter are limited in their ability to resolve crossing fibres in the brain, which impacts on the reliability of fibre-tracking (Farquharson et al. 2013). A number of approaches based on HARDI, including the constrained spherical deconvolution (CSD) technique (Tournier et al. 2007), allow multiple fibre orientations to be resolved. In adults, CSD approaches provide improved accuracy of tract delineation compared to DT based methods (Farquharson et al. 2013) and are able to delineate pathways connecting cerebellar hemispheres with contralateral cerebral cortex (Palesi et al. 2015).

The aim of this study was to assess the performance of these methods (HARDI data analysed with CSD) in delineating the CTC and CPC pathways in infants between 29 and 44 weeks postmenstrual age (PMA), and to assess the maturation of these pathways over this age range.

## Methods

Research Ethics Committee approval for MR imaging was granted by the West London National Research Ethics Committee (12/LO/1247) and written parental consent was obtained prior to MRI.

### Subjects

Inclusion criteria for this study were MR imaging with HARDI performed ≤44 weeks PMA. Exclusion criteria were congenital malformations, evidence of focal lesions on MRI in the cerebrum or cerebellum, or motion corrupted images (>5 dMRI volumes). Nine infants had >5 corrupt dMRI volumes and two had focal lesions on MRI [haemorrhage in posterior periventricular white matter (*n* = 1) and extensive punctate lesions throughout the white matter (*n* = 1)], and were excluded from subsequent analysis. Our final study group included twenty-four infants [15 male; 22 infants born preterm, <37 weeks gestational age (GA), and two healthy term controls]. The median GA of the infants was 33^+4^ (range 24^+6^–39) weeks and the median PMA at scan was 37^+1^ (29^+1^–44) weeks. Perinatal clinical details of the infants are described in Table 1.

**Table 1:** Perinatal clinical characteristics of the infants

Clinical characteristic	
GA at birth (median, range)	33^+4^ (24^+6^–39) weeks
PMA at scan (median, range)	37^+1^ (29^+1^–44) weeks
Days respiratory support (median, range)^a^	0 (0–134) days
Necrotising enterocolitis	4 infants
Small for gestational age^b^	8 infants

^a^Total days requiring mechanical ventilation, continuous positive airways pressure and supplementary oxygen

^b^Defined at <10th birthweight centile

### MR imaging

MR imaging was performed on a 3T Philips Achieva system (Best, The Netherlands) sited on the neonatal intensive care unit using a 32-channel head coil. 3D MPRAGE [repetition time (TR) = 17 ms, echo time (TE) = 4.6 ms, flip angle 13°, voxel size: 0.82 × 0.82 × 0.8 mm, scanning time of 7 minutes], T2 weighted fast spin echo (TR = 8670 ms, TE = 160 ms, flip angle 90°, slice thickness 2 mm with 1 mm overlapping slices, in-plane resolution 1.14 × 1.14 mm, scanning time of 4 minutes) and HARDI data were obtained. HARDI data were acquired in 64 non-collinear directions with b value of 2500 s/mm^2^, 4 non-diffusion weighted images (b0), resolution 2 mm isotropic, a SENSE factor of 2 and scanning time of 16 min. TR and TE were 9000 and 62 ms, respectively.

All examinations were supervised by a pediatrician experienced in MR imaging procedures. Pulse oximetry, temperature and electrocardiography data were monitored throughout. Ear protection was used, comprising earplugs moulded from a silicone-based putty (President Putty, Coltene Whaledent, Mahwah, NJ, USA) placed in the external auditory meatus and neonatal earmuffs (MiniMuffs, Natus Medical Inc., San Carlos, CA, USA). Preterm infants at term equivalent age were sedated with oral chloral hydrate (25–50 mg/kg) prior to scanning. Term controls and preterm infants <37 weeks PMA at scanning were not sedated.

### Data processing

All scans were visually inspected in three orthogonal planes prior to subsequent analysis and motion corrupted dMRI volumes were removed (only datasets with ≤5 motion-corrupted volumes were included). Diffusion data was corrected for eddy currents and motion using the eddy algorithm available within FSL 5.0 (Andersson and Sotirpoulos 2015a, b).

T2 images were anatomically parcellated in left and right cerebellar hemisphere and 90 supratentorial brain regions by a spatial non-rigid registration of the AAL neonatal brain atlas (Shi et al. 2011) using a consistent version (Tristan-Vega and Arribas 2007) of a block matching algorithm (Warfield et al. 2002). White and grey matter tissue segmentation was performed using a neonatal specific segmentation algorithm (Makropoulos et al. 2014). Following parcellation and segmentation, T2 weighted images were non-rigidly registered to the b0 image and anatomical labels were propagated onto the diffusion data.

### Tractography

Fiber orientation distributions (FOD) were estimated by CSD (Tournier et al. 2007, 2008) at each voxel using a harmonic order (*λ*_max_) of 8. Tractography was performed using seed, waypoint and target regions derived from the anatomical parcellations and manual delineation. Similar to previous studies (Palesi et al. 2015), to isolate the contralateral pathway it was necessary to place contralateral targets for the CTC and CPC tracts (Figure S1). To delineate the CTC tract, manually drawn seed regions were placed in the superior cerebellar peduncle where it enters the brainstem, with target regions consisting the whole of the ipsilateral cerebellar gray matter and contralateral cerebral cortical grey matter. Waypoint regions were contralateral thalamus and a manually delineated mid-sagittal section through the mesencephalon. To delineate the CPC tract, a manually delineated seed region was placed in the cerebral peduncle and target regions were contralateral cerebellar gray matter and ipsilateral cerebral gray matter with an exclusion mask in the mid-sagittal mesencephalon, forcing fibres to cross at the level of the pons. In this case an exclusion region was also placed in the thalamus. For each tract, 1000 streamlines were generated using probabilistic tractography available in MRtrix (Tournier et al. 2012). In addition, the DT model was also obtained for each subject, and fractional anisotropy (FA) of each voxel was calculated.

To examine connections between the cerebellum and contra-lateral cortex in more detail, tractography streamlines were analysed to establish which regions in the cortex defined by the AAL atlas were connected to contralateral cerebellum in each subject. This way, we reported which connections (≥10 streamlines) were reconstructed in most infants (≥75 % of cases) throughout this developmental period. The percentage of streamlines connecting each region was calculated by determining the ratio of streamlines connecting each AAL region over the total number of streamlines. The association between increasing PMA at scan and the average FA value and percentage of streamlines was calculated by means of partial Spearman’s correlations using GA at birth as a co-variable. Multiple comparisons were controlled by means of a false discovery rate (FDR) procedure, controlling alpha error to 5 % (Benjamini et al. 2006).

Statistical analysis was performed using SPSS 21.0 (SPSS, Chicago, IL) and MATLAB (2012b, The MathWorks Inc., Natick, MA). Computational algorithms were also implemented using MATLAB.

## Results

**Fig. 1:**
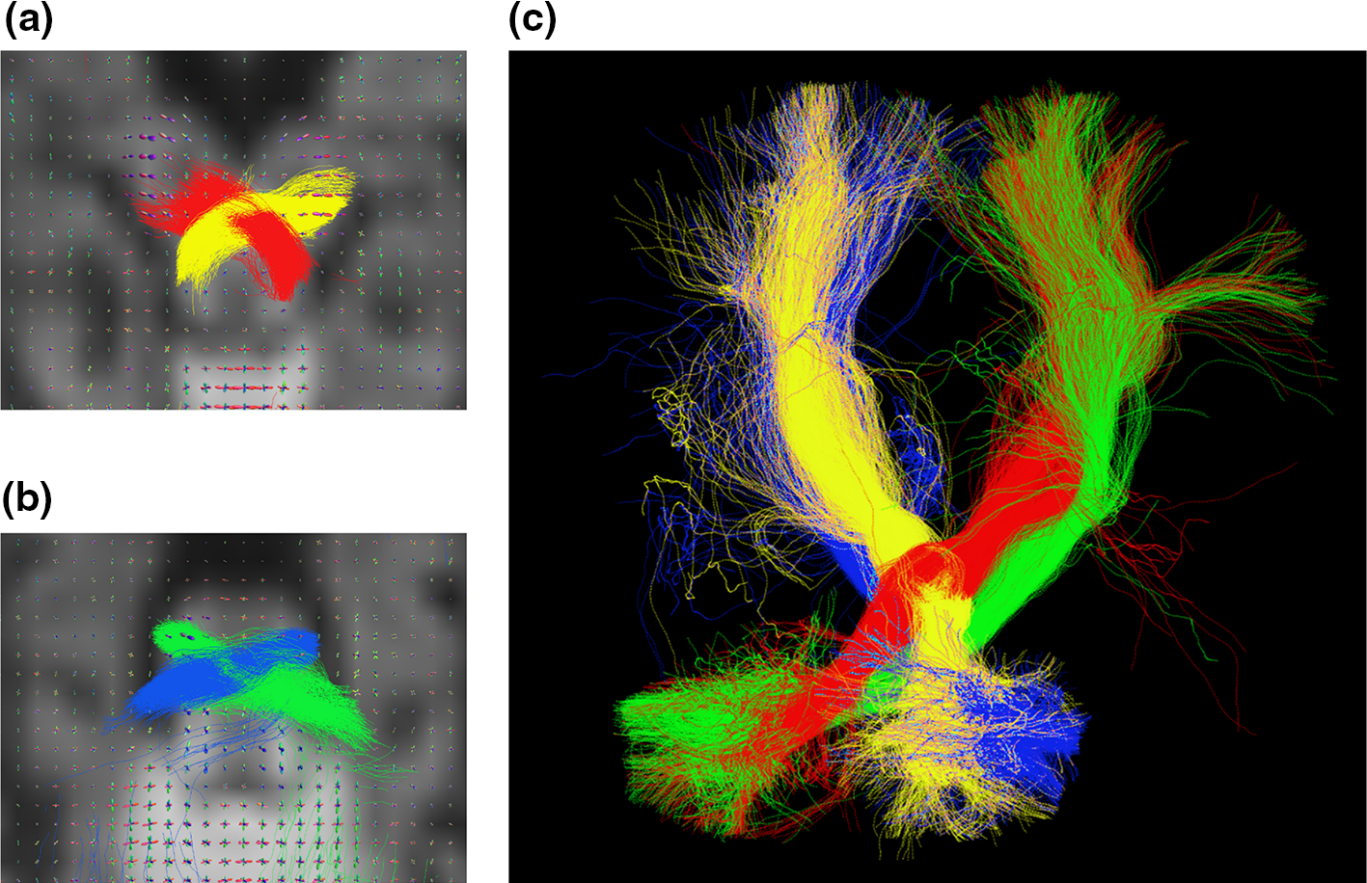
Reconstruction of cerebello-thalamo-cortical tract (CTC, *red-yellow*) and cortico-ponto-cerebellar tract (CPC, *blue-green*) in an infant born at 33 weeks and imaged at 40 weeks PMA with FOD plots overlaid on the diffusion data. **a** Crossing fibres of the CTC tract at the level of the mesencephalon. **b** Crossing fibres of the CPC tract at the level of the pons. **c** 3D reconstruction of both tracts

**Fig. 2:**
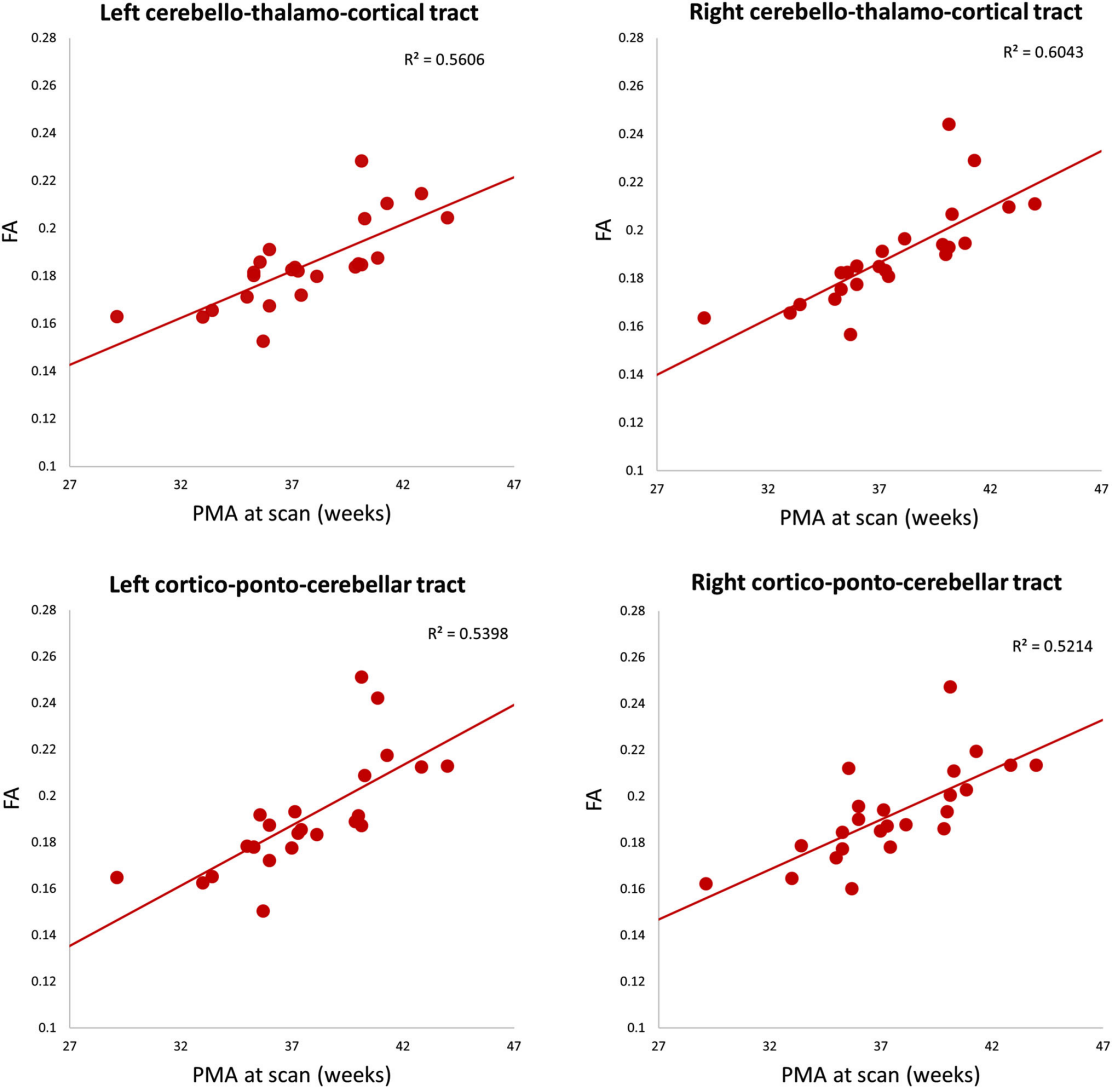
Fractional anisotropy values of whole cerebello-thalamo cortical tract (CTC) and cortico-ponto-cerebellar tract (CPC), plotted against PMA at scan (*horizontal axis*) for each subject

Coherent white matter pathways between cerebellar and cerebral cortices were reconstructed in all infants studied, suggesting that extensive white matter connections between cerebellum and cerebrum are already established at 29 weeks gestational age. We identified pathways passing through the superior cerebellar peduncle, crossing at the mesencephalon (Fig. 1a) and connecting to the contralateral thalamus and cerebral cortex (CTC tracts). Delineating pathways passing through the cerebral peduncle and connecting to contralateral cerebellum enabled visualisation of the CPC pathways (Fig. 1b). Figure 1c shows CTC and CPC pathways in a representative subject. FA values across the whole CTC and CPC pathways increased significantly with increasing PMA at scan (*p* < 0.001) (Fig. 2).

**Fig. 3:**
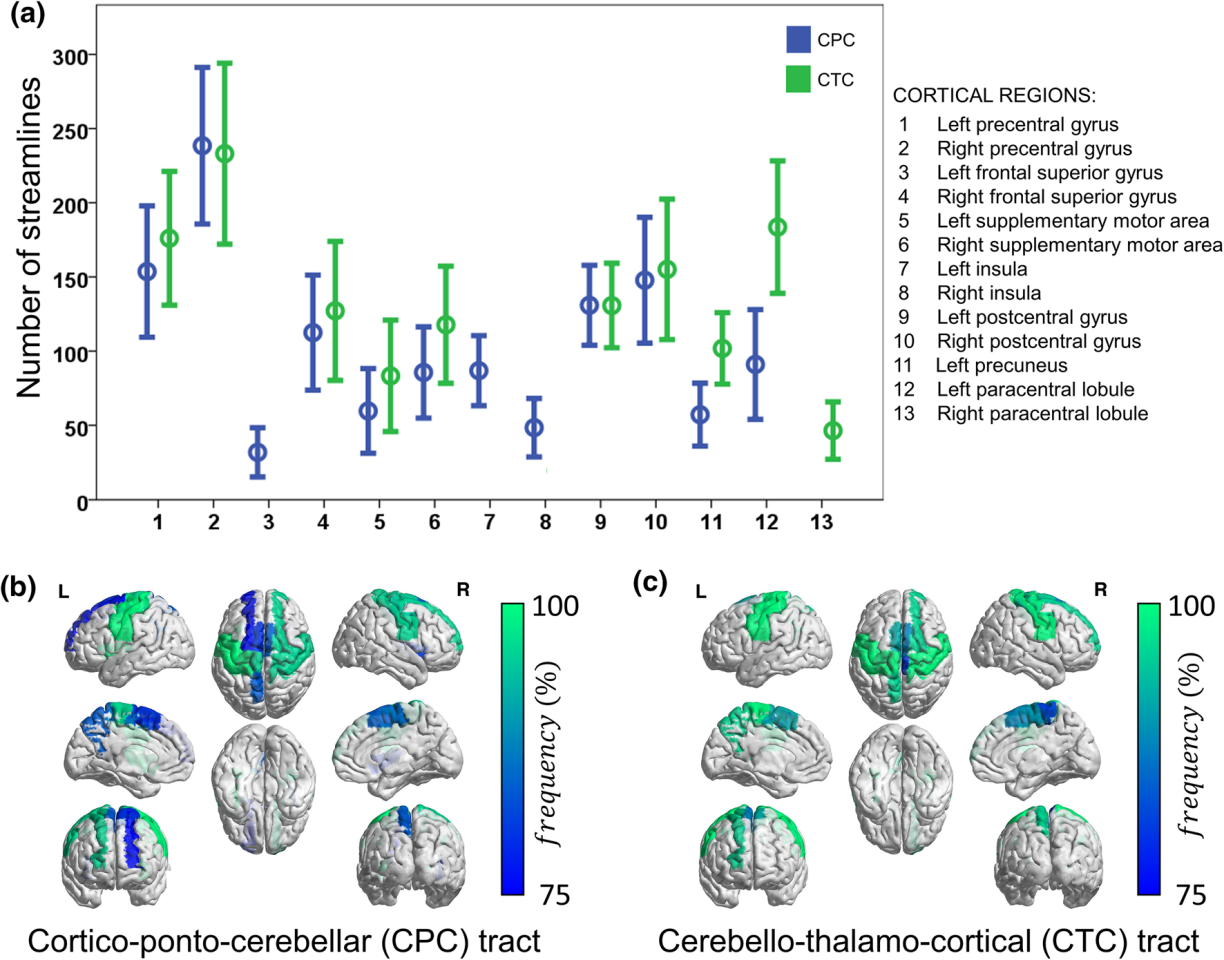
**a** Number of streamlines (mean and 95 % CI) reconstructed connecting cortical regions to contralateral cerebellar regions for cerebello-thalamo-cortical tract (CTC) and cortico-ponto-cerebellar tract (CPC). Cortical regions connected to contralateral cerebellar regions in at least 75 % of the subjects with more than ten streamlines through **b** CPC and **c** CTC tract. *Color map* shows frequency (%) of connections across the whole population

Using cortical parcellation, we were able to highlight regionally specific development of cerebro-cerebellar connectivity between the early preterm period and term equivalent. A set of cerebellar pathways connecting with supratentorial regions was reconstructed consistently (≥10 streamlines) for most (≥75 %) subjects irrespectively of age at scan (Fig. 3a; Table 2). For the CPC tracts, those regions were: the precentral cortex bilaterally, superior frontal cortex bilaterally, supplementary motor area bilaterally, insula bilaterally, postcentral cortex bilaterally, left precuneus, and left paracentral lobule (Fig. 3b). For the CTC tracts these regions included: precentral cortex bilaterally, right superior frontal cortex, supplementary motor area bilaterally, postcentral cortex bilaterally, left precuneus, and paracentral lobule bilaterally (Fig. 3c). We assessed the characteristics (age at MRI and GA at birth) of the infants in whom a specific tract was not reconstructed and observed that lower age at MRI was associated with an inability to delineate connections between left supplementary motor area and the CPC tract (*p* = 0.019, uncorrected). There was no relationship between age at scan or GA at birth and the ability to delineate any of the other cerebral-cerebellar connections (Supplementary Table 1).

**Table 2:** Percentage of infants in whom cerebello-cortical pathways (≥10 streamlines) were identified

Cortical region	CTC (%)	CPC (%)
Left precentral gyrus	100	100
Right precentral gyrus	100	96
Left superior frontal lobe	67	75
Right superior frontal lobe	96	96
Left supplementary motor area	88	79
Right supplementary motor area	83	79
Left insula	67	100
Right insula	50	79
Left postcentral gyrus	100	100
Right postcentral gyrus	100	96
Left precuneus	96	79
Left paracentral lobe	100	96
Right paracentral lobe	75	67

We also assessed the change in connectivity between 29 and 44 weeks PMA by assessing the correlation of the percentage of streamlines and average FA connecting cerebellum with different cortical regions through CPC and CTC tracts. For the CPC tracts, the percentage of streamlines connecting to right supplementary motor area [Spearman’s correlation coefficient (*ρ*) = 0.651, FDR-adjusted *p* = 0.030] increased significantly with PMA at scan. The average FA of the CPC tract connecting to the left postcentral gyrus (*ρ* = 0.595, FDR-adjusted *p* = 0.033) was positively correlated with PMA at scan.

For the CTC tracts, the percentage of streamlines connecting to the left supplementary motor area (*ρ* = 0.588, FDR-adjusted *p* = 0.025) was positively correlated with PMA at scan and the percentage of streamlines connecting to the right postcentral gyrus (*ρ* = −0.580, FDR-adjusted *p* = 0.025) was negatively correlated with PMA at scan.

## Discussion

In this study we demonstrate the feasibility of delineating the CTC and CPC tracts in infants as young as 29 weeks gestational age in vivo. The CTC and CPC pathways are multi-synapse and characterized by a high degree of convergence and divergence (fanning) along their trajectory. Furthermore, between the cerebellar and cerebral cortex, tracts pass through micro structurally complex regions, including crossing axonal fascicles at the level of the brainstem (Arnts et al. 2014). Delineating connections between cerebellar and cerebral cortex using diffusion tractography is, therefore, challenging and to our knowledge there have been no previous studies in the developing brain in vivo. The CSD based probabilistic approach used here, in combination with HARDI data, was able to reconstruct pathways that corresponded well to anatomical dissections (Arnts et al. 2014; Naidich et al. 2008; Perrini et al. 2013) and tracer studies of these tracts (Angaut et al. 1985; Green and Wingate 2014; Martin et al. 1987; Sawyer et al. 1994; Teune et al. 2000).

We identified connections between the cerebellum and a wide range of supratentorial brain regions, including both motor and non-motor domains. Throughout this developmental period, connections were identified between the cerebellum and supratentorial brain regions thought to be mainly involved in motor coordination, including the primary motor cortex, supplementary motor area, precentral gyrus and postcentral gyrus (Chouinard and Paus 2006; Hardwick et al. 2013; Potgieser et al. 2014). However, connections were also identified between the cerebellum and the superior frontal gyrus and insula. The superior frontal gyrus is considered to play a role in cognition, self-awareness, working memory, attention and language processing (Cutini et al. 2008; du Boisgueheneuc et al. 2006; Goldberg et al. 2006; Kamali et al. 2014a, b; Koenigs et al. 2009; Tully et al. 2014; Wang et al. 2015). The insular cortex is believed to play a major role in consciousness, emotion and perception of pain and auditory signals (Bamiou et al. 2003; Brooks et al. 2005; Craig 2009; Sander and Scheich 2005; Steinbrink et al. 2009; Uddin 2015). These results are in line with the results of Palesi et al. (2015), in which CSD-based tractography suggested prominent connectivity of CTC fibers to non-motor cortical areas in adults (Palesi et al. 2015).

Previous dMRI studies assessing the developing brain have shown increases in FA in supratentorial white matter in the period prior to normal term birth (Braga et al. 2015; Bui et al. 2006; Gao et al. 2009; Huppi et al. 1998; Kersbergen et al. 2014), consistent with increased fibre density. Elegant ex vivo studies from 17 weeks GA have shown similar findings in the white matter pathways of the cerebellum (Takahashi et al. 2014). We observed that FA values across the whole of the CPC and CTC pathways increased with increasing maturity. As these pathways pass through multi-synaptic nuclei, FA values in these grey matter regions will be included in the measured FA, but the proportion of voxels containing largely grey matter will be relatively small compared to voxels containing white matter fibres. Furthermore, we were able to depict changes in connectivity between 29 and 44 weeks PMA by assessing the percentage of fibres and average FA of streamlines to distinct cortical areas. Significant correlations were observed with average FA of CPC streamlines connecting to left postcentral gyrus and with the percentage of streamlines connecting CPC and CTC tracts with right and left supplementary motor area, respectively, suggesting regional variation in microstructural changes during this period. The sample size in this study was relatively small, and we used stringent corrections for multiple comparisons. Further studies including more infants may identify significant maturational changes in additional CTC and CPC pathways. We are aware of limitations of assessing streamline count (Jones et al. 2013) and so chose to assess our results as a percentage of streamlines in the CTC and CPC pathways.

There are some limitations to our study, which are inherent to all studies using dMRI to characterise neuroanatomy and have been succinctly described recently (Palesi et al. 2015). Despite validation studies showing that diffusion characteristics correspond well with white matter alignment (Gao et al. 2013; Hubbard et al. 2015; Seehaus et al. 2013), the technique is not comparable to invasive axonal tracing and related techniques. Delineating multi-synaptic pathways, including the CPC and CTC, is challenging and the sharp turning-angle of the CPC at the level of the pontine nuclei is problematic for most tracking algorithms, which typically operate with an assumption of low streamline curvature. Indeed, as in previous studies (Palesi et al. 2015), both ipsi- and contra-lateral pathways were delineated when contralateral waypoints were not included in the protocol. We addressed this issue using a probabilistic tractography algorithm, in combination with strong constraints imposed on the tractography by placing ROIs based on prior anatomical knowledge to depict these pathways. The optimal *b* value and number of diffusion weighted gradients for robust estimation of multiple fibre directions in the neonatal brain have not been defined. Simulation studies using adult parameters suggest that *b* values between 2200 and 2800 s/mm^2^ are optimal for fibre orientation estimation (Alexander and Barker 2005) and increasing *b* values beyond 3000 s/mm^2^ does not improve angular contrast to noise ratio in vivo (Tournier et al. 2013). While a minimum of 45 independent gradient directions are required to fully characterize the diffusion weighted signal at this *b* value in adults, acquiring more than this minimum number is recommended to increase signal to noise ratio (Tournier et al. 2013). In this study we used a *b* value of 2500 s/mm^2^ and 64 non-collinear directions; however, further work is required to determine the optimal *b* value and number of distinct directions required for HARDI in neonates.

Cerebellar dysmaturation and injury is associated with a wide range of neuromotor, neurocognitive and behavioral disorders including ASD (Wang et al. 2014; Wegiel et al. 2014), ADHD (Berquin et al. 1998; Tomasi and Volkow 2012; Wang et al. 2013) as well as with preterm birth. For ASD, specific alterations in cerebellar anatomy have been identified (Carper and Courchesne 2000; Courchesne et al. 1988; D’Mello et al. 2015; Fatemi et al. 2012), and the presence of cerebellar injury at birth is suggested to be the largest single non-heritable risk factor for developing ASD (Wang et al. 2014). In infants diagnosed with ADHD, altered cerebellar activity (Kucyi et al. 2015; Wang et al. 2013) and connectivity (Takahashi et al. 2010; Tomasi and Volkow 2012; van Ewijk et al. 2012) patterns have been identified using resting state fMRI and dMRI, as well as anatomical cerebellar abnormalities (Berquin et al. 1998; Bledsoe et al. 2011; Stoodley 2014).

The pre- and early postnatal periods are considered crucial for morphogenesis, growth and differentiation (Volpe 2009) and the cerebellum is vulnerable to disrupted development during this critical timeframe (Limperopoulos et al. 2005a, 2009; Ranger et al. 2015; Volpe 2009; Zayek et al. 2012), putting preterm-born infants at risk for developing neurodevelopmental sequelae related to disrupted cerebellar microstructure (Haines et al. 2013; Limperopoulos et al. 2005b, 2007; Messerschmidt et al. 2008). Characterizing cerebellar microstructure and development during this developmental process may help clarify injurious mechanisms that give rise to disrupted cerebellar structure and function. The impact of aberrant cerebellar output on motor and non-motor functions, for example, remains to be elucidated. However, there is increasing evidence for crossed cerebellar diaschisis, that is impaired functional connectivity and cerebellar growth, in infants with unilateral supra-tentorial lesions (Limperopoulos et al. 2005c). In addition, MRI studies in preterm infants have highlighted impaired development of the contralateral cerebral hemisphere following cerebellar injury that is largely confined to cerebral regions activated by afferent pathways from the contralateral cerebellum (Limperopoulos et al. 2010). Of note, cerebellar injury in this population leads not only to impaired growth of remote cerebral cortical regions but also to domain-specific functional impairments (Limperopoulos et al. 2014). There is, therefore, a pressing need for approaches that are able to characterize non-invasively cortico-cerebellar and cerebello-cortical development.

In summary, in this study we have demonstrated the feasibility of delineating cortico-ponto-cerebellar and cerebello-thalamo-cortical tracts in vivo during the early stages of development, using HARDI in combination with the CSD based probabilistic tractography. The ability to assess normal and impaired cerebro-cerebellar connectivity during this critical period will help elucidate the impact of cerebellar lesions on connections to the cerebrum and improve our understating of the role of the cerebellum in a wide range of pervasive neuromotor and neurocognitive disorders.

## Electronic supplementary material

Below is the link to the electronic supplementary material.

Figure S1. Cerebello-thalamo-cortical pathways in two infants who were imaged at 40 weeks PMA (a and b) and 29 weeks PMA (c and d). The seed ROI was placed in the left superior cerebellar peduncle. (a and c) No target ROI was included. (b and d) Target regions consisting the whole of the ipsilateral cerebellar gray matter and contralateral cerebral cortical grey matter, with waypoint regions in the contralateral thalamus and a mid-sagittal section through the mesencephalon (TIFF 13331 kb)

Supplementary material 2 (DOCX 17 kb)
