# Supplementary figures and images for: Cerebello-cerebral connectivity in the developing brain
Source: Brain Struct Funct. 2016 Aug 29;222(4):1625–34. doi: 10.1007/s00429-016-1296-8 (PMC5406415; doi:10.1007/s00429-016-1296-8)

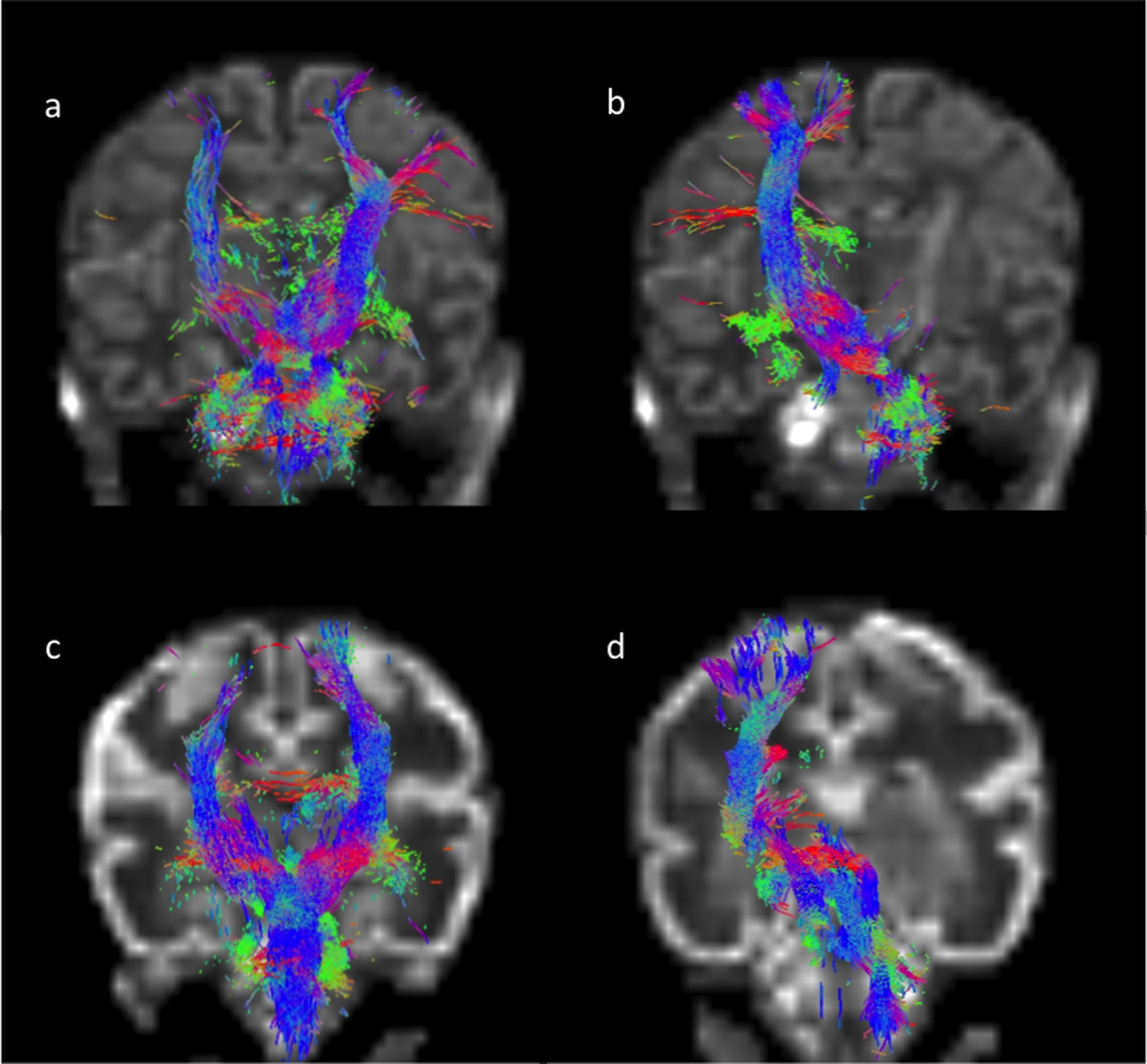

Supplement: Supplementary file 1 — Figure S1. Cerebello-thalamo-cortical pathways in two infants who were imaged at 40 weeks PMA (a and b) and 29 weeks PMA (c and d). The seed ROI was placed in the left superior cerebellar peduncle. (a and c) No target ROI was included. (b and d) Target regions consisting the whole of the ipsilateral cerebellar gray matter and contralateral cerebral cortical grey matter, with waypoint regions in the contralateral thalamus and a mid-sagittal section through the mesencephalon (TIFF 13331 kb) [file 429_2016_1296_MOESM1_ESM.tif]
