# Supplementary material for: Cerebello-cerebral connectivity in the developing brain
Source: Brain Struct Funct. 2016 Aug 29;222(4):1625–34. doi: 10.1007/s00429-016-1296-8 (PMC5406415; doi:10.1007/s00429-016-1296-8)
Supplement: Supplementary file 2 — Supplementary material 2 (DOCX 17 kb) [file 429_2016_1296_MOESM2_ESM.docx]

**Supplementary Table 1.** Age at MRI and GA at birth of infants in whom cerebello-cortical pathways (≥10 streamlines) were identified. Results of GLM assessing differences in GA at birth and age at MRI are reported when significant (p < 0.05).

| **Cortical region** | CTC (%) | Mean (SD) age at MRI [weeks]  (subjects where tract was identified versus those where the tract was not) | Mean (SD) GA at birth [weeks]  ( subjects where tract was identified versus those where the tract was not ) | CPC (%) | Mean (SD) age at MRI [weeks]  ( subjects where tract was identified versus those where the tract was not) | Mean (SD) GA at birth [weeks]  ( subjects where tract was identified versus those where the tract was not) |
| --- | --- | --- | --- | --- | --- | --- |
| Left precentral gyrus | 100 | - | - | 100 | - | - |
| Right precentral gyrus | 100 | - | - | 96 | - | - |
| Left superior frontal lobe | 67 | 37.4 (2.9) vs. 37.8 (4.7) | 33.8 (3.0) vs. 31.4 (4.5) | 75 | 37.3 (3.6) vs. 38.3 (3.8) | 33.0 (3.8) vs. 33.5 (3.1) |
| Right superior frontal lobe | 96 | - | - | 96 | - | - |
| Left supplementary motor area | 88 | - |  | 79 | **38.4 (3.0) vs. 34.4 (3.6) p=0.019** | 33.0 (3.6) vs. 33.4 (3.6) |
| Right supplementary motor area | 83 | 37.8 (3.5) vs. 35.4 (0.5) | 33.9 (3.7) vs. 34.4 (1.1) | 79 | 37.8 (3.4) vs. 36.0 (3.9) | 33.0 (3.9) vs. 33.5 (2.9) |
| Left insula | 67 | 36.9 (3.5) vs. 39.1 (2.7) | 33.5 (3.9) vs. 34.6 (2.3) | 100 | - | - |
| Right insula | 50 | 38.8 (2.6) vs. 36.2 (3.7) | 33.0 (3.5) vs. 33.2 (3.8) | 79 | 38.2 (2.9) vs. 36.0 (3.8) | 33.15 (3.8) vs. 33.0 (3.6) |
| Left postcentral gyrus | 100 | - | - | 100 | - | - |
| Right postcentral gyrus | 100 | - | - | 96 | - | - |
| Left precuneus | 96 | - | - | 79 | 37.6 (3.5) vs. 37.3 (3.6) | 32.9 (3.6) vs. 34.2 (3.6) |
| Left paracentral lobe | 100 | - | - | 96 | - | - |
| Right paracentral lobe | 75 | 37.6 (2.7) vs. 37.4 (5.9) | 33.6 (3.7) vs. 31.2 (1.9) | 67 | 37.4 (2.5) vs. 37.8 (3.3) | 34.0 (3.3) vs. 31.0 (3.4) |
